# Complex gene expression in the dragline silk producing glands of the Western black widow (*Latrodectus hesperus*)

**DOI:** 10.1186/1471-2164-14-846

**Published:** 2013-12-02

**Authors:** Amanda Kelly Lane, Cheryl Y Hayashi, Gregg B Whitworth, Nadia A Ayoub

**Affiliations:** 1 Department of Biology, Washington and Lee University, 204 W. Washington St., Lexington, VA 24450, USA; 2 Biology Department, University of California Riverside, Riverside, CA 92521, USA

**Keywords:** Spidroin, Major ampullate glands, Alternative polyadenylation, MaSp1, Spider, Tag profiling

## Abstract

**Background:**

Orb-web and cob-web weaving spiders spin dragline silk fibers that are among the strongest materials known. Draglines are primarily composed of MaSp1 and MaSp2, two spidroins (spider fibrous proteins) expressed in the major ampullate (MA) silk glands. Prior genetic studies of dragline silk have focused mostly on determining the sequence of these spidroins, leaving other genetic aspects of silk synthesis largely uncharacterized.

**Results:**

Here, we used deep sequencing to profile gene expression patterns in the Western black widow, *Latrodectus hesperus*. We sequenced millions of 3′-anchored “tags” of cDNAs derived either from MA glands or control tissue (cephalothorax) mRNAs, then associated the tags with genes by compiling a reference database from our newly constructed normalized *L. hesperus* cDNA library and published *L. hesperus* sequences. We were able to determine transcript abundance and alternative polyadenylation of each of three loci encoding MaSp1. The ratio of *MaSp1*:*MaSp2* transcripts varied between individuals, but on average was similar to the estimated ratio of MaSp1:MaSp2 in dragline fibers. We also identified transcription of *TuSp1* in MA glands, another spidroin family member that encodes the primary component of egg-sac silk, synthesized in tubuliform glands. In addition to the spidroin paralogs, we identified 30 genes that are more abundantly represented in MA glands than cephalothoraxes and represent new candidates for involvement in spider silk synthesis.

**Conclusions:**

Modulating expression rates of MaSp1 variants as well as MaSp2 and TuSp1 could lead to differences in mechanical properties of dragline fibers. Many of the newly identified candidate genes likely encode secreted proteins, suggesting they could be incorporated into dragline fibers or assist in protein processing and fiber assembly. Our results demonstrate previously unrecognized transcript complexity in spider silk glands.

## Background

Spiders (Araneae) use silk throughout their lives for functions such as prey capture, sperm webs, egg cases, safety draglines, and dispersal [1]. Orb-web weavers and their relatives (Orbiculariae) spin six or more task-specific silk fibers. Each of these task-specific fibers is synthesized in its own set of specialized abdominal glands [1,2]. For example, dragline silk is synthesized in the major ampullate (MA) glands and egg-case silk is synthesized in the tubuliform glands. Each type of silk has a unique suite of impressive mechanical properties [3]. The dragline silk of orb-weavers approaches the tensile strength of steel and is more extensible than rubber or tendon collagen [4,5]. This combination of strength and extensibility makes dragline silk incredibly tough. Tubuliform silk, by contrast, is a stiff fiber that can store a large amount of energy [3]. There is interest in capitalizing on the remarkable variation and combination of material properties of spider silks for medical and industrial applications such as tendon replacements, sutures, and as an environmentally friendly alternative to synthetic fibers [6,7].

Spider silks are composed primarily of large structural proteins termed spidroins (spider fibrous proteins) that are encoded by members of a single gene family. Silk types differ from each other in their constituent spidroins [8,9]. For instance, MA glands express MaSp1 and MaSp2 [10,11] and the outer egg case silk synthesized in tubuliform glands includes TuSp1 [12-14]. Other spidroin paralogs have been identified from each of the gland types except for the aggregate gland [15-18]. Spidroins are large proteins (200-350 kDa, e.g. [19-23]) made up almost entirely of repeated blocks of amino acids (aa), flanked by non-repetitive amino (N) and carboxy (C) terminal domains. These terminal domains are conserved in length (~150 aa) and sequence among spidroin types and across species [22,24-26].

The majority of spider silk genetic research has focused on silk synthesized in the MA glands because of the high tensile strength of dragline fibers [4,6,7]. These efforts include characterizing *MaSp1* and *MaSp2* sequences from multiple species (e.g^.^[9,22]) and creating recombinant proteins expressed in transgenic organisms (e.g. [27,28]). However, the mechanical properties of fibers spun from recombinant spider silk proteins do not yet accurately mimic natural spider silk fibers (e.g. [29,30]), suggesting more extensive work is needed to understand the genetics and spinning of spider silks. Synthesis, assembly, and material properties of dragline silk likely rely on the relative expression of spidroin paralogs as well as the influence of non-spidroin genes. For instance, the relative proportion of MaSp1 and MaSp2 in dragline silk varies among species and even among individuals of the same species, which could potentially explain inter-and intra-specific level differences in mechanical properties [31-33]. The extensibility of dragline silk correlates positively with its proline content [32,33], an amino acid that is present in higher proportion in MaSp2 than in MaSp1 [11]. The Western black widow, *Latrodectus hesperus* (Theridiidae), synthesizes draglines with a low proline content and thus MaSp1 is predicted to be approximately three times more abundant than MaSp2 in the fiber [22,34]. The extensibility of black widow dragline silk is correspondingly lower than other species that have draglines primarily composed of MaSp2 [33]. Additionally, MaSp1 is encoded by multiple loci in *L. hesperus* and the golden orb-weaver *Nephila clavipes*[35,36]. *MaSp1* loci are similar to each other, but not identical, within a species. Variation in the expression patterns of each locus could contribute to variation in mechanical properties of dragline silks within or among species, but such variation in expression has yet to be documented.

Non-spidroin genes can also play important roles in the synthesis and assembly of spider silks, although only a few cases have been described (e.g. [37,38]). For instance, the egg case proteins, ECP-1 and ECP-2, are expressed primarily in the tubuliform glands and are predicted to form cross-links with TuSp1 [37]. Exploring the genetics of spider silk synthesis has been limited to a gene-by-gene approach because of the minimal genomic information available for spiders. Genomic methods have only recently become feasible for non-model organisms, such as spiders, with the advent of next generation sequencing technologies [39-41]. Next generation sequencing and *de novo* assembly were used to characterize 18,000 “uni-genes” expressed in the silk glands of two spider species, a tarantula and an orb-weaver [42]. This approach identified several major functional classes of genes expressed in silk glands, and discovered new spidroin paralogs, but did not measure relative expression rates. These results represent a major advance in characterizing spider genes, but due to the lack of comparisons with other tissues, it is unclear which genes or functional classes are important for silk synthesis [42].

Here, we use massively parallel signature sequencing (MPSS) to profile expression patterns in MA glands and cephalothoraxes (fused head-body) of the Western black widow. We targeted this species for several reasons. Black widows synthesize dragline silks that are among the strongest measured [5,43]. As descendants of orb-web weaving ancestors [44], black widows possess all the gland types of true orb-web spiders despite building three-dimensional cobwebs [45]. Six of the seven known spidroin paralogs have been described for the Western black widow. Furthermore, because the Western black widow has a relatively small genome for a spider (~1.3 billion base pairs [46]), we have arrayed a genomic library and completely sequenced the dragline silk genes *MaSp1* and *MaSp2*[22] and the prey-wrapping gene *AcSp1*[47]. Our aims were to 1) determine relative levels of transcript abundance within MA glands compared to cephalothorax control tissue and 2) identify novel candidate genes of importance to dragline silk production. Our findings demonstrate that dragline silk synthesis involves a much greater transcriptional complexity than previously known, laying the foundation for further studies of silk gland functional genomics and recombinant silk production.

## Results and discussion

### Generation of “tags” and construction of reference database

We generated more than 20 million tags, which are 3′-anchored 20 base cDNA fragments, from mRNA transcripts found in the MA glands and cephalothoraxes of adult female Western black widows using MPSS (Additional file 1: Figure S1; e.g. [48-51]). MPSS allows for highly quantitative comparison between tissues, even in organisms with poorly characterized genomes, by anchoring sequencing to the 3′ end of transcripts. Different transcripts are discriminated by their 3′-most tags and expression levels estimated with higher accuracy than could be achieved by random RNA-Seq [52]. Sequencing more than 5 million tags from each of four libraries (two made from MA glands and two from cephalothoraxes) resulted in 200,603 unique tag sequences. Of these, 32,111 unique tags were found at levels greater than one count per million total tags (cpm) in two or more libraries (Table 1).

**Table 1:** Counts of tags generated from two major ampullate (MA) gland and two cephalothorax cDNA libraries

	**Tags sequenced > 1 time**	**Tags with > 1 cpm**^**a **^**in at least 2 libraries**	**Normalization factor**
MA silk gland 1	6,626,230	6,392,914	1.01
MA silk gland 2	6,731,324	6,346,320	1.08
Cephalothorax 1	6,587,276	6,316,307	0.87
Cephalothorax 2	5,912,202	5,648,579	1.05
Total unique tags	200,603	32,111	

^a^cpm = counts per million.

To determine the gene identity of tags, we compiled a non-redundant set of 526 “reference” protein-coding sequences for the Western black widow from 263 newly sequenced cDNAs [GenBank: GW787472-787523;GW820091-800100;JZ531018-531200], 343 published cDNA sequences, and 25 published gene sequences (mitochondrial sequences were excluded from construction of reference database). The only sequences available that included untranscribed regions were for the complete gene sequences of *MaSp1* and *MaSp2*. For initial expression profiling, we thus limited representatives of *MaSp1* and *MaSp2* to the longest cDNA for each.

The MPSS method we used targeted the 3′-most occurrence of the *DpnII* recognition sequence, 5′-GATC-3′. We determined tag identity by first predicting all possible tag sequences from the coding or sense strand and the complementary or antisense strand (GATC and 16 following bases) in the reference genes. Predicted tags were then matched to the sequenced tags from our four libraries. Of the 515 sequences in our reference database (when *MaSp1* and *MaSp2* are represented by one cDNA each), 409 contained a predicted tag and 200 were represented by at least one observed tag.

### Identification of differentially expressed transcripts in Western black widows

**Figure 1:**
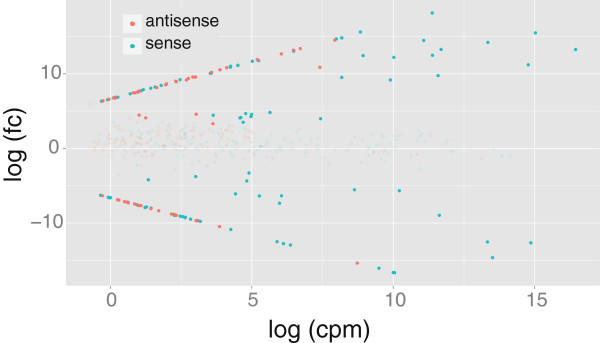
**Tag abundance versus log fold change, log (fc), between major ampullate (MA) glands and cephalothoraxes.** Tags included are only those that matched a reference gene. Tag abundance, log (cpm), is the natural log of average counts of that tag per million total tags (cpm) among all four libraries. Tags that are differentially expressed between MA glands and cephalothoraxes (FDR ≤ 0.05) are bolded. Turquoise tags matched the sense strand and salmon tags matched the antisense strand. A positive log (fc) indicates higher abundance in MA glands.

An exact test implemented in edgeR [53] identified approximately 23% of tags were significantly more abundant in one tissue type: 2,323 unique tags were significantly more abundant in the MA glands and 5,010 tags were more abundant in the cephalothoraxes (false discovery rate, FDR ≤ 0.05). Each of these differentially expressed tags was at least 1000 times more abundant in one tissue than the other (Additional file 2: Table S1). We assigned 386 unique tags to 200 genes in our reference database (Additional file 1: Figure S2). In terms of abundance and fold change, all tag sequences that matched a reference gene (Figure 1) as well as tags that matched the 3′-most position (Additional file 1: Figure S3) appear to be an unbiased subset of all observed tags. Abundance levels of these tags were also highly correlated between individual samples (Additional file 1: Figure S4).

**Figure 2:**
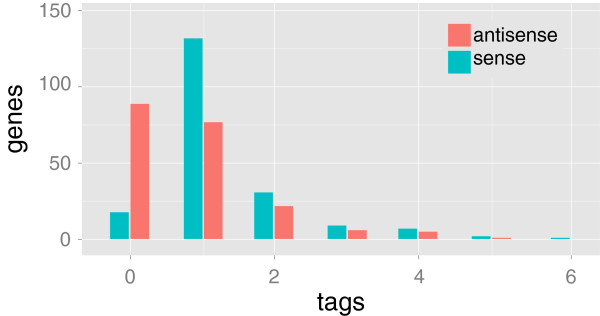
**Numbers of black widow reference genes represented by a given number of tags.** Tags that matched the sense strand are shown in turquoise and tags that matched the antisense strand are shown in salmon.

Approximately 46% of the reference genes represented by a sequenced tag were represented by both sense and antisense tags, 45% were represented by only a sense tag, and 9% were represented by only an antisense tag (Figure 2 and Additional file 1: Figure S2). Most reference genes were represented by one sense tag and/or one anti-sense tag (Figure 2; average sense tags/gene = 1.325, range = 0-6; average antisense tags/gene = 0.82, range = 0-5; average total tags/gene = 2.145, range = 1-9).

We observed instances of multiple tags aligning to a single gene, but the 3′-most sense tag was almost always considerably more abundant than other sense tags, as expected (Additional file 1: Figure S5). Genes represented by multiple sense tags were usually represented by multiple antisense tags, but antisense tags did not show a consistent relationship between position on the gene and tag abundance (Additional file 1: Figure S5). Alternative tags could result from incomplete digestion with *DpnII*, or from degradation of mRNAs. However, in many cases multiple tags represent genuine alternative transcripts (e.g. alternatively spliced or polyadenylated forms). An example of these alternative transcripts is described for *MaSp1* below. Because the origin of multiple tags per gene is not always clear, we estimated transcript abundance two ways. First, under the assumption that all tags that matched a gene represent transcription of some kind, we summed the average cpm of all tags from the sense strand, or separately, from the antisense strand (Additional file 1: Figure S6a). Second, to account for the possibility that multiple tags represent alternative transcripts, we estimated transcript abundance based solely on the 3′-most position of the sense strand, or its matched position on the antisense strand (Additional file 1: Figure S6b). These two estimates are tightly correlated (Additional file 1: Figure S7).

Because we selected for polyadenylated mRNA, we did not expect to capture antisense transcripts. Total abundance of a transcript estimated from summing all tags that matched that gene sequence is correlated between sense and antisense strands, with total abundance of sense tags almost always higher than total abundance of antisense tags (Additional file 1: Figure S6a). For instance, expression levels based on sense tags varied from 0 to 164,899 cpm in MA glands while antisense counts varied from 0 to 1,287 cpm. Despite lower abundance of antisense tags, differences in gene expression levels between tissues estimated by sense and antisense tags are tightly correlated (Additional file 1: Figure S8). Thus, even if our antisense tags represent an experimental error associated with the reverse transcription phase of cDNA synthesis [54], they provide consistent information regarding differential expression.

It is also possible that antisense transcription is widespread in spiders. In metazoan genomes investigated thus far, 5-22% of genes are represented by both sense and antisense transcripts [55]. In mice and humans, more than 40% of the transcript pool could result from antisense transcription [54]. Furthermore, expression patterns of sense and antisense transcripts generated from the same genomic locus tend to be correlated [56], similar to the pattern seen here (Additional file 1: Figure S8).

### Variable expression of spidroin paralogs in MA glands

**Figure 3:**
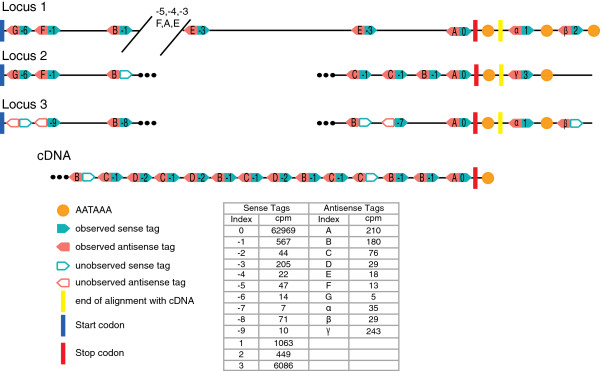
**Diagram of all predicted tags for three *MaSp1 *loci and the longest *MaSp1 *cDNA.** Observed (filled arrows) and unobserved (empty arrows) tag locations are approximate and not to scale. Antisense (salmon) and sense (turquoise) tags are paired by location. These *MaSp1* tags are not predicted for any other gene in the reference database. Dots (•••) indicate missing sequence data. Slashed lines in Locus 1 indicate ~5 kilobases of sequence data that is not shown. Observed unique tag indexes are numbered for sense tags (0 being 3′-most tag in the protein coding sequence and -9 being 5′ most) and lettered for antisense tags (A being the 3′-most of the protein coding sequence and G being the 5′-most). Observed tags that appear after the stop codon are labeled with positive integers (sense indices 1, 2, 3) and Greek letters (antisense indices α, β, γ). Tag abundance is the average counts of that tag per million total tags (cpm) in the two major ampullate libraries. Multiple occurrences of some tag sequences reflect the repetitive nature of *MaSp1*. Start codon (blue hatch), stop codon (red hatch), and the polyadenylation signal AATAAA (orange dot) are indicated. The cDNA aligns with the three genomic loci up to the yellow hatch.

Our database contained 11 unique sequences encoding MaSp1, including one complete protein-coding sequence for locus 1 and several thousand base pairs of non-coding flanking sequence [22]. It additionally included representatives of two other loci (loci 2 and 3) and partial length cDNAs. From these sequences, including non-coding regions, we predicted 123 sense tags (66 unique) and 122 antisense tags (59 unique). Full-length *MaSp1* has 11 predicted sense tags (8 unique) and 11 predicted antisense tags (7 unique) that matched an observed tag. Importantly, all of the tags predicted in the coding region of the full-length gene were observed as well as two downstream of the coding region (Figure 3). As expected, there were no observed tags that corresponded to intergenic genomic DNA. In addition to the eight unique sense tags that matched the full-length *MaSp1* gene, there were five other unique sense tags that aligned to alternative *MaSp1* sequences (Figure 3).

Alignment of all *MaSp1* sequences indicated that the three loci and the cDNAs share an identical observed sense tag in the C-terminal encoding region (Index 0, Figure 3). This tag is the 3′-most predicted one in the cDNAs and was the most abundant observed *MaSp1* tag and the second most abundant observed tag in MA glands overall (mean cpm in MA glands = 62,969; mean cpm in cephalothoraxes = 1.5; Figure 3). The seven sense tags found throughout the repetitive encoding region of the full-length *MaSp1* were much less abundant (mean cpm in MA glands = 14-567), consistent with selecting for the 3′-most occurrence of the *DpnII* recognition sequence.

Although the 3′-most tag from the coding sequence cannot discriminate expression levels of each *MaSp1* locus, there are other indices that are diagnostic for particular loci (Figure 3). Prior to this study, only locus 2 was supported by expression data [35]. Here, we observed unique tags for all three loci. Locus 2 has an observed unique tag 482 bases downstream of the stop codon (Index 3, Figure 3; mean cpm in MA glands = 6086). Loci 1 and 3 share an observed tag 144 bases downstream of the stop codon that is not found in locus 2 (Index 1, Figure 3; mean cpm in MA glands = 1063). Yet another observed unique tag occurs in locus 1, 486 bases downstream of the stop codon (Index 2, Figure 3; mean cpm in MA glands = 449). A different tag was predicted in the same location in locus 3, but was not observed. However, locus 3 has a unique observed tag in the N-terminal coding region (Index = -9, Figure 3; mean cpm in MA glands = 10). Thus, we have evidence for the co-expression all three loci at the same time in the same tissue.

The observed tags found downstream of the *MaSp1* stop codon also suggest that both loci 1 and 2, and possibly 3, are alternatively polyadenylated. Consistent with this hypothesis is the occurrence of a polyadenylation signal (AATAAA) downstream of an observed tag in each of these loci (Figure 3). Both 3′ UTR length, and specific sequence elements contained therein, have been shown to be important for a variety of regulatory events including control of mRNA stability and translational efficiency [57]. Our results thus raise the possibility that differential polyadenylation of *MaSp1* transcripts may be involved in locus-specific stability or translational control.

Although the 3′-most coding region tag of *MaSp1* is identical among all three loci (Index 0, Figure 3), the sequences of downstream tags are unique and can therefore be used to compare locus-specific expression levels. Analysis of these tags reveals that the isoform of locus 2 carrying an elongated 3′ UTR is ~4 times more abundant than the alternatively polyadenylated forms of loci 1 and 3 combined (6086 cpm vs. 1512 cpm, respectively; Figure 3). In the absence of strong, locus-specific alternative 3′ end regulation, this finding suggests that locus 2 is dominant in the MA gland tissue of adult females. Our data are therefore consistent with the previously suggested hypothesis that MaSp1 locus variants could be incorporated in differing proportions into draglines [35]. Because MaSp1 encoded by each locus differs in proportion and ordering of ensemble repeats, varying the proportion of each could affect silk properties. Importantly, prior to this study there was no direct evidence for simultaneous expression from all three loci.

Our database also contained seven sequences encoding MaSp2, including a complete gene with several thousand base pairs of non-coding flanking sequence [22]. From these *MaSp2* sequences, 184 tag locations (64 from the MaSp2 coding region) were predicted from the sense strand representing 100 unique tag sequences (29 from the MaSp2 coding region). In the full-length gene, 33 tag locations were observed (11 unique tag sequences). Four of these observations were well outside the coding region and appeared at low frequencies in all tissues (cpm < 6.3), leaving seven unique observed sense tags within the MaSp2 coding region (Additional file 1: Figure S9). Alignment of the complete *MaSp2* gene with other *MaSp2* sequences including partial cDNAs showed that all sequences shared a 3′-most predicted sense tag. This tag was the most abundant in *MaSp2* sequences (mean cpm in MA glands = 18,386). All other observed *MaSp2* tags were much less abundant (mean cpm in MA glands = 2-68). There was only one additional unique sense tag observed from other *MaSp2* sequences. This tag is in the N-terminal encoding region of a partial *MaSp2* cDNA (mean cpm in MA glands = 4.39). A tag that only differed by one base pair from this tag was predicted from the corresponding coding region in the full-length gene, but was not observed (Additional file 1: Figure S9).

The ratio of *MaSp1*:*MaSp2* transcript abundance based on the 3′-most coding tags varied between the two individuals, but the ratio based on the average cpm in MA glands is 3:1 *MaSp1*:*MaSp2* consistent with the amino acid composition of MA fibers collected from Western black widows [22,34]. Thus, despite the potential for complex locus-specific translational control, transcript abundance may accurately reflect overall protein abundance of MaSp1 and MaSp2.

Variation in proportion of MaSp1 and MaSp2 could explain variation in fiber properties among individual black widow spiders [58]. In three other species, varying the amount of protein in the spider’s diet significantly alters mechanical properties of MA fibers [59]. Specifically, extensibility of MA fibers, a property highly correlated with the proline content of the fiber [32,33], decreased with a low protein or protein-less diet [59]. Since proline is in higher proportion in MaSp2 than in MaSp1 [11], an increased extensibility in MA fibers is likely due to an increase in the proportion of MaSp2 in the fiber. Individual spiders could have widely varying diets that overtime could change the proportion of spidroins in the silk fibers and thus create fibers with varying mechanical properties.

**Figure 4:**
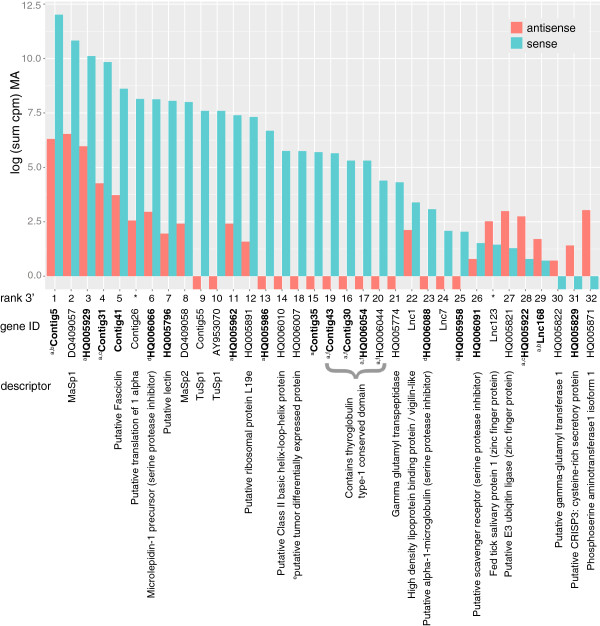
**Expression levels of black widow reference genes.** Total sense (turquoise) and antisense (salmon) transcript abundance for genes with an observed tag more abundant in major ampullate (MA) glands than cephalothoraxes (FDR ≤ 0.05). Transcript abundance is the sum of the average log counts per million (cpm) of all tags that matched to that gene between two MA libraries. Reference genes are ranked according to decreasing total abundance. Rank abundance according to just the 3′-most tag (rank 3′) is almost identical (see also Additional file 1: Figure S6c). Genes that were not significantly different according to the 3′-most tag are marked with an asterisk (*). Descriptor is the top BLASTX hit to NCBI non-redundant (nr) protein database. Bolded genes have a signal peptide predicted by SignalP using D-value cut off = “sensitive”. ^a^Only BLASTX hits are hypothetical proteins in *L. hesperus*. ^b^Contig5 and Lnc168 are nearly identical for the first 178 bases. Tags are located downstream in regions that do not align between the two sequences. ^c^Contig31 and HQ005922 align with 42% identity over 43 amino acids. ^d^HQ006066 and HQ006098 align with 47% identity over 54 amino acids. ^e^Aligns to hypothetical proteins from multiple species using BLASTX. ^f^These four sequences align with ~73-95% pairwise identity over the entire amino acid sequence (81-93 amino acids). Contig43, Contig30 and HQ006054 share a tag. Contig43 and HQ006044 share an additional tag. HQ006044 has a predicted tag in the same location as the other three, but it is not observed.

*TuSp1*, which encodes the primary component of outer egg-cases, was unexpectedly expressed in MA glands. Our non-redundant database contained two sequences predicted to encode TuSp1, but both shared an identical observed tag in the C-terminal encoding region (mean cpm in MA = 996; mean cpm in cephalothorax = 0). *TuSp1* was surprisingly abundant, ranking 10^th^ most represented gene of the ones that are more abundant in MA glands than cephalothoraxes, directly following *MaSp2* (Figure 4). However, one individual transcribed much more *TuSp1* (1990 cpm) than the other (2 cpm). The difference in expression levels between the two individuals could be explained by individual differences in readiness to make egg-cases. The number of transcripts encoding TuSp1 and other egg-case proteins increase in tubuliform silk glands as the individual nears egg-case production [60]. The individual with lower levels of TuSp1 in MA glands did not have well-developed eggs. However, the individual with high levels of *TuSp1* had large, mature eggs, suggesting that *TuSp1* transcription increases in MA glands at the same time as increasing in tubuliform glands. It is possible TuSp1 becomes incorporated in MA fibers at the time of egg-case production, which could contribute to variation in mechanical properties. Alternatively, translational control of *TuSp1* prevents protein synthesis in MA glands despite the presence of *TuSp1* transcripts. We also cannot exclude the possibility that our detection of *TuSp1* in MA glands is due to leakage from mRNAs transcribed in the tubuliform glands.

### Novel candidate genes involved in silk synthesis

In addition to spidroin encoding genes, we identified 30 (28 when analysis is limited to the 3′-most tags) other genes represented by tags that were significantly more abundant in MA glands than cephalothoraxes (FDR ≤ 0.05; Figure 4). When translated, 20 (18 when analysis is limited to the 3′-most tags) of these genes aligned to functionally annotated proteins or conserved domains (Figure 4). Of the remaining ten genes, one did not match any entries in the NCBI nr database and nine aligned only to hypothetical proteins previously predicted for Western black widows. All but one of these ten encode proteins with signal peptides predicted by SignalP [61] (Figure 4), suggesting they are secreted. Secreted proteins could be components of MA fibers or involved with fiber assembly.

An unanticipated finding was that the 3′-most sense transcript tag of one of the hypothetical protein-encoding genes is three times more abundant in MA glands than that of *MaSp1* (Figure 4). This gene, “Contig5”, has a short (45 aa) open reading frame (ORF) and SignalP predicts an even smaller mature peptide (19 aa). The first 178 bases, which includes the first 27 aa, of “Contig5” is nearly identical to that of another gene overrepresented in MA glands, “Lnc168”, as well as one (“Lnc106”) that was not differentially expressed between MA and cephalothorax. Each of these sequences differs in the last few amino acids and most of the putative 3′ UTRs, including the tag locations. “Lnc168” is much less abundant in MA glands than “Contig5” (Figure 4). These sequences may represent transcripts from paralogous genes, alternatively spliced genes, or a combination of both scenarios. Alignment revealed two regions of almost exact identity among all three sequences, and another region of exact identity between two, interspersed with unalignable regions. These patterns are consistent with both hypotheses of paralogous genes and alternatively spliced genes. Alternative splicing is an important mechanism for generating functionally distinct proteins with tissue specific expression [62] as well as unique 3′ UTRs. Isoforms caused by 3′ UTR alternative splicing can modify the production of the protein through altering locations for microRNA binding and activating decay pathways such as the nonsense-mediated decay pathway [63,64].

Among the most highly expressed genes in MA glands that could be annotated were ones predicted to encode fasciclin, translation elongation factor 1-alpha, and lectin. Each of these was more abundant than *MaSp2* (Figure 4). Abundance ranking of genes expressed in MA glands was almost identical if estimates were based solely on the 3′-most tags (Figure 4; Additional file 1: Figure S6c). Tags corresponding to *elongation factor 1-alpha* were only slightly more abundant in MA glands than cephalothoraxes (log fold change = 1-3.5). In fact, the 3′-most sense tag of this gene was not significantly different between the two tissues. Thus, the higher abundance in MA glands probably reflects the very high rates of translation to produce dragline spidroins, MaSp1 and MaSp2. In contrast, tags representing fasciclin and lectin-encoding genes were consistently highly abundant in MA glands but either absent or extremely low in cephalothoraxes (log fold change = 9.7-14.5). In various insect species, fasciclins are cell adhesion glycoproteins that play a role in neuronal development (e.g. [65]). Lectins are sugar-binding proteins involved in the innate immune response in *Drosophila*[66,67]. However, it is not immediately clear how these particular functions might be involved with silk synthesis. It is possible that these genes are expressed in specialized cells within the MA glands that are not secreting silk proteins. The MA glands are divided into multiple regions including a tail and ampullate region where spidroins are expressed, the lower ampullate region where spidroins are stored, and the duct where spidroins begin assembly for ultimate extrusion as fibers [20,68]. There may well be cells within any of these regions that have currently unknown functions not found in the cephalothorax.

Thirteen of the 20 genes overrepresented in MA glands that were annotated by sequence similarity are predicted to encode proteins that can be grouped into three functional classes (Figure 4). These include protease inhibitors (three genes align to serine protease inhibitors and four align to cysteine protease inhibitors), proteins with transferase activity (two genes align to gamma-glutamyltransferases and one to phosphoserine aminotransferase), and transcription factors (three genes, or two if analysis is restricted to 3′-most tags, align to conserved DNA binding domains found in transcription factors and thus could serve to increase transcription of spidroins and other silk gland specific proteins). The protease inhibitors appear to be derived from two large gene families. The putative serine protease inhibitor genes encode 54 aa domains that align to each other with 47% pairwise identity. They also align to four additional translated genes in our reference database that were not overrepresented in MA glands. Similarly, four of the genes overrepresented in MA glands encode thyroglobulin type-1 conserved domains, which function as cysteine protease inhibitors. These four genes align with each other with 85-89% pairwise nucleotide identity over their entirety. At the amino acid level, they align to an additional four sequences in our reference database. The numerous protease inhibitors expressed in MA glands likely reflect the importance of maintaining protein integrity in the storage compartment of the MA glands. Widow spiders regularly spin dragline silk and need a ready supply of functional spidroins to incorporate into fibers. These upregulated gene family members may be specialized for maintaining spidroins.

One of the genes predicted to encode a protein with transferase activity is homologous to a phosphoserine aminotransferase, which is part of the cascade for serine biosynthesis as shown in *Escherichia coli*[69]. Another two transcripts encode gamma-glutamyltransferases also known as gamma-glutamyltranspeptidases (γ-GT), which in mammals, are primarily utilized in the glutathione pathway [70]. This pathway is implicated in protection against oxidative stress and redox regulation of gene expression [70]. Because spider MA silk processing involves a series of pH changes in the gland [68], oxidation regulation is likely to be crucial and could be mediated by γ-GT-like proteins.

## Conclusions

Through cDNA tag profiling, we describe previously unrecognized gene expression complexity in MA glands of the Western black widow. As expected, *MaSp1* and *MaSp2* were among the most highly expressed genes in MA glands. Surprisingly, the most abundant transcript in MA glands was one with unknown function. Also unexpected, was the discovery of high levels of *TuSp1* expression and an additional six genes that were more abundantly represented in MA glands than *MaSp2* (Figure 4). In addition, we demonstrated the simultaneous, but unequal expression of three *MaSp1* loci. Alternative polyadenlyation of *MaSp1* and alternative splicing of other genes upregulated in MA glands may also increase the complexity of individual variation of MA proteins. We propose that modulating the composition of MaSp1 variants, MaSp2, and possibly TuSp1 within silk glands will alter the material properties of dragline silk. Hence, if the ratios of these spidroins change as a consequence of ontogeny, physiology, or the environment, this can contribute to variation in the properties of silks spun by the same spider (intraindividual) or different (intraspecific) spiders.

We identified 34 unique gene sequences represented by tags that were significantly more abundant in MA glands than cephalothoraxes. Besides spidroin genes, we found 30 new candidate genes for dragline silk synthesis. Approximately 1/3 of these genes have no known homolog outside of *Latrodectus*. It is possible that these genes resulted from black widow specific evolutionary events. However, due to the paucity of genomic resources for spiders we cannot exclude the possibility that these genes have homologs in other spiders. Identifying homologs expressed in silk glands of other spider species would strengthen the argument that these genes are involved in silk synthesis. Of the genes predicted to encode proteins with functional homologs some may simply reflect the high rates of translation and protein storage in silk glands such as translation factors, amino transferases, and protease inhibitors. Others are likely transcription factors important for regulating gland-specific spidroin expression. Gamma-glutamyl-transferases may be important regulators of pH changes in the MA gland that are necessary for fiber assembly. An overriding theme for many of the genes overrepresented in MA glands is that they are members of larger gene families. Neofunctionalization of gene copies expressed in silk glands may be even more important for silk synthesis than the previously recognized diversity of the spidroin gene family. As such, silk glands represent a model system for understanding the evolution of tissue specific functions. Furthermore, our increased understanding of gene regulation in spider silk glands will ultimately lead to improved recombinant silk production.

## Methods

### Construction and sequencing of “tag” libraries

Major ampullate (MA) glands and cephalothoraxes were dissected from two *Latrodectus hesperus* adult females caught live in Riverside, California (USA). Total RNA was isolated from each tissue type for both individuals (four separate isolations) by homogenizing tissue in TRIzol**®** (Invitrogen, Carlsbad, CA) and further purifying RNA with the RNeasy Mini Kit (Qiagen, Valencia, CA). Any remaining genomic DNA was removed by incubating RNA with DNase from the Ambion® TURBO DNA-*free*™ kit (Invitrogen).

We then constructed four cDNA “tag” libraries using the DGE: tag profiling for *DpnII* kit (Illumina, Inc., San Diego, CA). In brief, we extracted single stranded poly(A) mRNA using Sera-mag magnetic oligo(dT) beads. Single stranded DNA complementary to the mRNA (cDNA) was synthesized using SuperScript**®** III Reverse Transcriptase (Invitrogen), primed from the oligo(dT) magnetic beads. Still attached to the magnetic beads, second strand cDNA was synthesized by incubation with RNaseH and DNA Polymerase I. The double stranded cDNA was digested with *DpnII*, which recognizes 5′-GATC-3′. The digested cDNA was washed and only the portion of cDNA still attached to the magnetic bead was retained. An adapter was ligated to the site of *DpnII* digestion, introducing the recognition sequence for *MmeI*. The adapter-cDNA ligation was digested with *MmeI*, which cuts 20 bp downstream from the recognition sequence and 16 bp downstream of the *DpnII* site, GATC. A second adapter was then ligated to the *MmeI* cut site, and the adapter-ligated cDNA construct was enriched by PCR and purified (Additional file 1: Figure S1). The resulting libraries were each sequenced in a separate lane of a flow cell on the Genome Analyzer II DNA Sequencer (Illumina) at the University of California Riverside Genomics Core Instrumentation Facility.

### Generation of reference database

In order to describe novel protein-coding genes from Western black widows, we constructed a normalized cDNA library. Total RNA was extracted and pooled from three adult females by homogenization in TRIzol® (Invitrogen) followed by purification with the RNeasy Mini Kit (Qiagen). mRNA isolation, cDNA synthesis, normalization, library construction, and arraying were performed by BIO S&T (Montreal, Canada). In brief, cDNA was synthesized from 5 μg of mRNA using the SMART™ cDNA library construction kit (Clontech, USA). Abundant cDNAs were reduced in frequency by subtractive hybridization. This method hybridizes a sample of biotyntilated single stranded cDNA to excess single stranded cDNA copies in the library. Double stranded cDNA is then removed using streptavidin beads. This allows for more efficient library sequencing. Following normalization, cDNAs were amplified, subjected to *SfiI* digestion, and size fractionated in a 1% agarose gel. Fragments greater than 0.5 kilobases were purified for cloning. cDNA fragments were ligated into a modified pBluescript II SK(-) vector between *EcoRI* (5′) and *XhoI* (3′) and used to transform bacterial cells (*Escherichia coli* strain DH10B from Invitrogen). Over 18,000 recombinant clones were arrayed into 48 384-well plates. Clones were sequenced using M13R or T3 to obtain the 5′ sequence of cDNAs (62 previously submitted to NCBI’s EST database and 201 newly submitted for this study; Additional file 3: Table S2). Two clones were sequenced with an oligo(dT)-V primer to obtain sequence from the 3′ end of cDNAs.

All 368 published *L. hesperus* nuclear protein-coding sequences were downloaded from the NCBI nucleotide database in June 2012 and combined with the ESTs. We generated a non-redundant set of sequences using CAP3 [71], which resulted in 50 contiguous sequences (contigs) and 476 singletons. These 526 sequences represented our reference database for assigning “tags” to genes. We identified homologs of these sequences using BLASTX [72] (universal genetic code, default parameters, e-value cut off 10^-6^) comparisons to NCBI’s non-redundant protein database (nr). We predicted signal peptides from translated genes using SignalP 4.1 [61], with D-cutoff value set to “sensitive”. We input the longest ORF starting with a methionine unless BLAST predicted an alternative ORF.

### Sequence processing and identification of differentially expressed “tags”

Illumina sequence reads were converted to fastq files including the base call and quality scores. Adapter and low quality sequences were removed with utilities in Bioconductor’s [73] Biostrings package, version 2.24.1 [74] (Additional file 1). Unique 16 base “tags” were identified and counted within each library. Any tags sequenced only once were removed. Tag counts were proxies for the expression level of the associated genes.

We analyzed tag counts from the four libraries with edgeR [53]. We filtered out low abundance tags retaining only tags with a count per million (cpm) of greater than one in at least two of the four libraries [50]. We generated multi-dimensional scaling plots of the libraries using plotMDS [75] and applied TMM (trimmed mean of M) normalization to account for the differences in library sizes and composition [76]. The exact test for the negative binomial distribution was used to compare the tag counts from the cephalothorax to the MA libraries [77]. The common dispersion and tagwise dispersion across all libraries were estimated [77,78]. The common dispersion is the squared coefficient of variation which gives the amount of variability in the abundance of each tag between replicate libraries, whereas the tagwise dispersion measures this variation for each individual tag rather than the total pool. The false discovery rate (FDR) was estimated using the Benjamini and Hochberg [79] algorithm. We considered genes represented by a tag with an FDR ≤ 0.05 to be differentially expressed between MA glands and cephalothoraxes.

### Matching observed tags to genes in reference database

To match our observed tags to genes in the reference database, we determined the locations and sequences of possible tags from our reference genes in both the sense and antisense directions. We ensured that all input sequences represented the coding strand (or sense transcript). First, we assumed that all sequences from NCBI’s nucleotide database had been correctly annotated. Second, our normalized cDNAs were positionally cloned so that sequencing with M13R or T3 resulted in characterizing the 5′ end of coding sequences. Clones sequenced from the 3′ end of the cDNA were reverse complemented prior to searching for tag sequences. Third, we inspected contigs assembled by CAP3 for retention of directionality. If a contig was reverse complemented relative to the raw sequences, we reverse complemented the contig. Finally, we checked that the coding frames predicted by BLASTX were all positive.

We batch imported our FASTA file of reference gene sequences into an R data frame and identified the tag positions by searching for “GATC” (Additional file 1). The position information generated above was used to retrieve the 16 bases following the “GATC” to generate the predicted tags from the sense strand. Similarly, 16 bases preceding the “GATC” were retrieved and reverse complemented to generate the predicted tags from the antisense strand. We then merged observed tag counts and statistics from the edgeR analysis with the table of predicted tags to obtain expression information for genes in our reference database.

### Relative expression levels of transcripts

We estimated expression levels of transcripts represented by genes in our database by summing counts for all unique tags that matched that gene sequence. Tags that aligned to more than one location in a sequence were only counted once. Because *MaSp1* and *MaSp2* were each represented by multiple non-redundant gene and cDNA sequences, we restricted analysis of expression levels to the longest cDNA associated with each gene [GenBank:DQ409057 and GenBank:DQ409058 respectively]. We also estimated expression levels from only the counts of the 3′-most tag of the sense strand, or the matched position for antisense abundance.

## Abbreviations

MA: Major ampullate; MPSS: Massively parallel signature sequencing; CPM: Counts per million; ORF: Open reading frame; FDR: False discovery rate.

## Competing interests

The authors declare they have no competing interests.

## Authors’ contributions

AKL analyzed the data and drafted the manuscript. CYH helped conceive the experimental design, helped collect data, and revised the manuscript. GBW analyzed the data and revised the manuscript. NAA conceived the experiments, collected data, contributed to analytical design, and helped draft the manuscript. All authors read and approved the final manuscript.

## Supplementary Material

Additional file 1 Contains Supplementary figures S1-S9 and supplementary methods. Click here for file

Additional file 2: Table S1 Summary statistics for 32,111 unique tags that were sequenced at least one time for every 1 million total tags sequenced (≥ 1 CPM) in at least two libraries. Tag Sequence gives the 16 bases that were sequenced. GATC was added to the 5′ end of these sequences for matching tags to genes. Abundance of tags in each library is given as counts per million (CPM) for two individuals (1 or 2) from cephalothoraxes (ceph) or major ampullate glands (MA). Log Fold Change (log FC) is between MA and cephalothoraxes. Log CPM is the log of the average CPM among all four libraries. P-values and the estimated False Discovery Rate (FDR) are associated with an exact test implemented in edgeR for differential expression between MA and cephalothoraxes. Click here for file

Additional file 3: Table S2 Summary information for genes in our reference database that matched an observed tag. geneID gives the GenBank accession number or the CAP3 contig number. (Accession numbers for ESTs generated in this study will be made available upon acceptance of manuscript). Tag sequence is the 16 bases that were sequenced. GATC was added to the 5′ end of these sequences for matching tags to genes. Gene descriptor gives the top BLASTX hit for contigs and newly generated ESTs or the gene descriptor from GenBank. Tag index describes placement of tag along gene, with 0 representing the 3′-most tag and smaller numbers moving progressively toward the 5′ end. Position is the base number of the sequence at which the G of GATC begins. Gene length is the number of bases in the sequence and FDR is the False Discovery Rate from the exact test for differential expression between major ampullate glands and cephalothoraxes (see Additional file 2: Table S1). Click here for file
