# Supplementary material for: Complex gene expression in the dragline silk producing glands of the Western black widow (Latrodectus hesperus)
Source: BMC Genomics. 2013 Dec 2;14:846. doi: 10.1186/1471-2164-14-846 (PMC3879032; doi:10.1186/1471-2164-14-846)
Supplement: Additional file 1 — Contains Supplementary figures S1-S9 and supplementary methods. [file 1471-2164-14-846-S1.pdf]

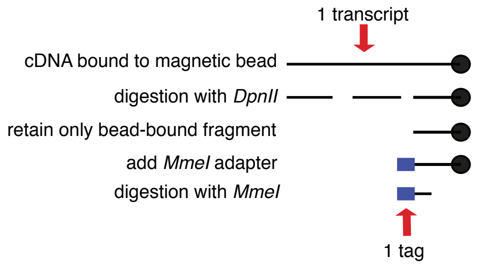

Supplementary Figure S1. Generation of tags for massively parallel signature sequencing (MPSS). Double-stranded cDNA is synthesized on magnetic oligo-dT beads. cDNA is digested with *DpnII* followed by magnetic separation to retain only the region after the most 3’ *DpnII* site. Adapters for *MmeI*, a restriction enzyme that cuts 20 bp downstream of its recognition sequence, are added. After *MmeI* digestion, the 20 bp tag represents the cDNA transcript.

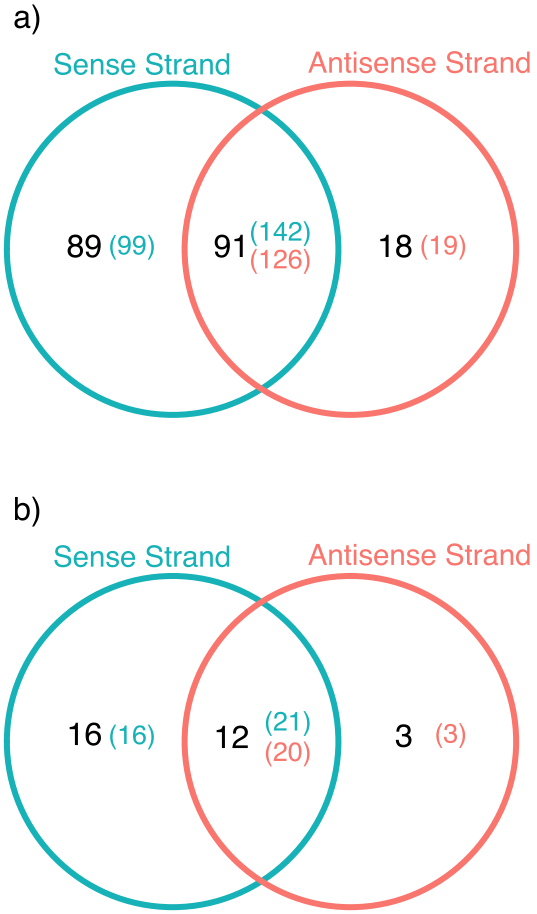

Supplementary Figure S2. Sense and antisense tag representation for genes in black widow reference database. (A) Total number of genes (black number) represented by a tag that matched the sense strand (number of tags in parentheses in turquoise) or a tag that matched the antisense strand (number of tags in parentheses in salmon). Overlapping section of Venn diagram indicates the number of genes (black) represented by both a sense tag and an antisense tag. (B) The number of unique gene sequences represented by tags (number of tags in parentheses) significantly more abundant in major ampullate (MA) glands than cephalothoraxes (FDR ≤ 0.05), excluding *MaSp1* and *MaSp2*. Overlapping section of the Venn diagram indicates the number of genes (black) that are more abundant in MA glands that are represented by both a sense tag and an antisense tag. If the analysis of differential expression is limited to the 3'-most sense tags, then two genes are lost from the overlapping section.

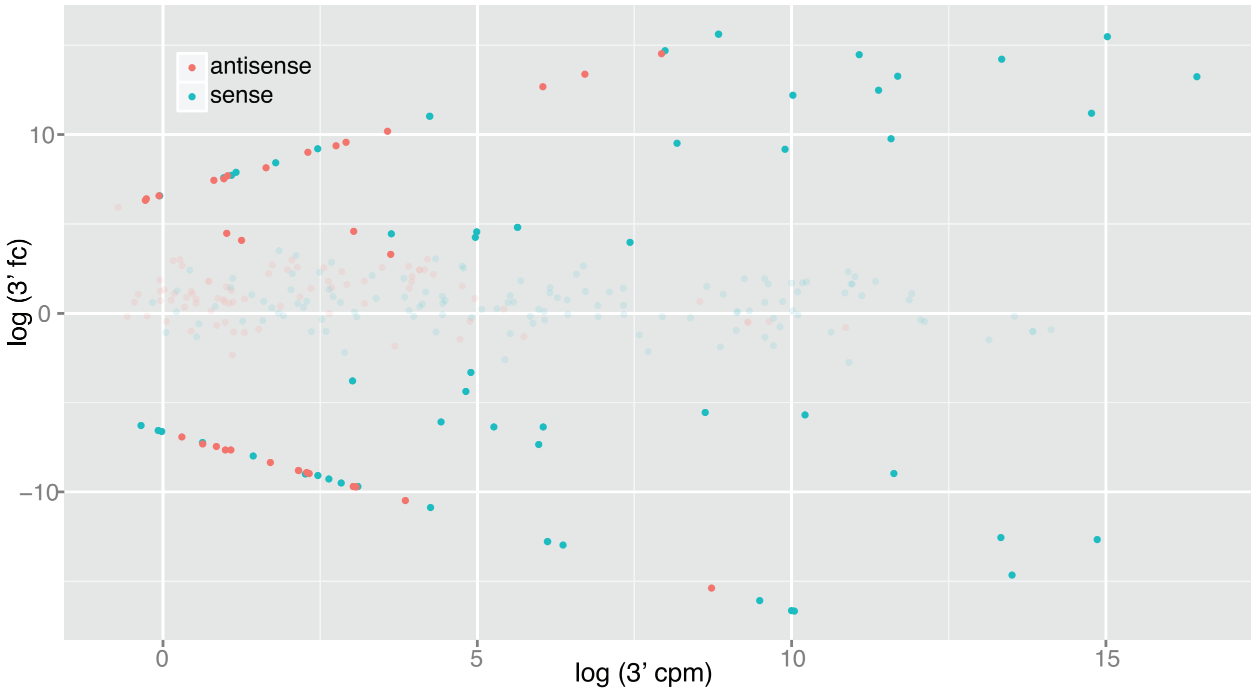

Supplementary Figure S3. Tag abundance versus log fold change between major ampullate (MA) glands and cephalothoraxes. Tags included are only those that matched the 3'-most position of the coding strand of a reference gene. Tag abundance, log (3' cpm), is the natural log of average counts of that tag per million total tags (cpm) among all four libraries. Tags that are differentially expressed between MA glands and cephalothoraxes (FDR ≤ 0.05) are bolded. Turquoise tags matched the sense strand and salmon tags matched the antisense strand. A positive fold change, log (3' fc), indicates higher abundance in MA glands.

a)

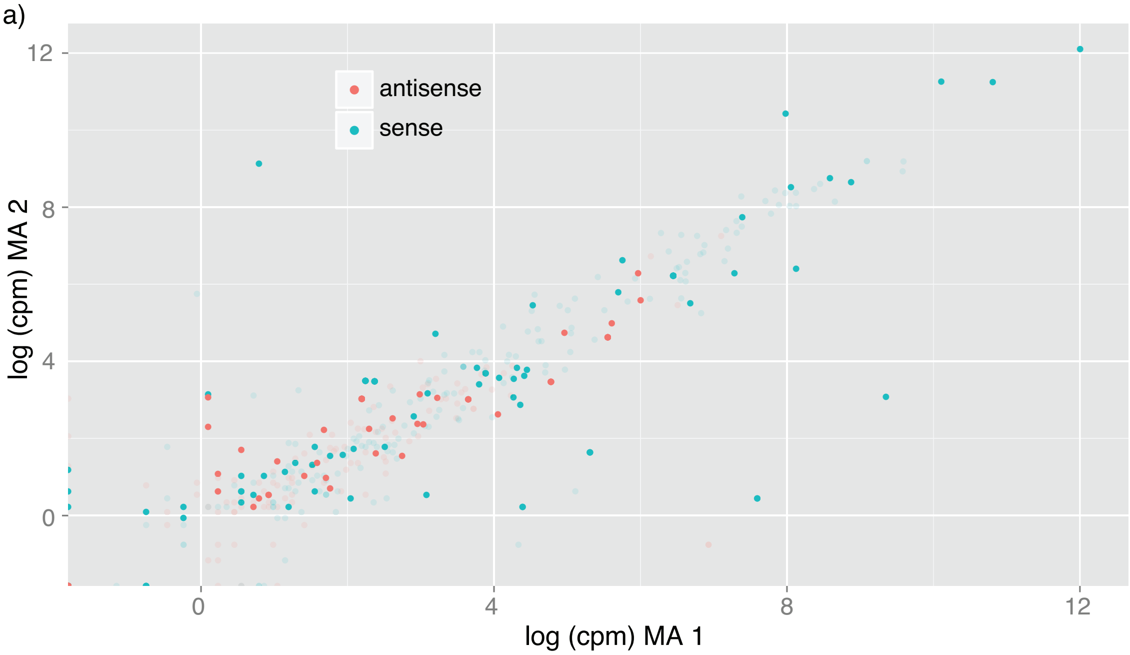

b)

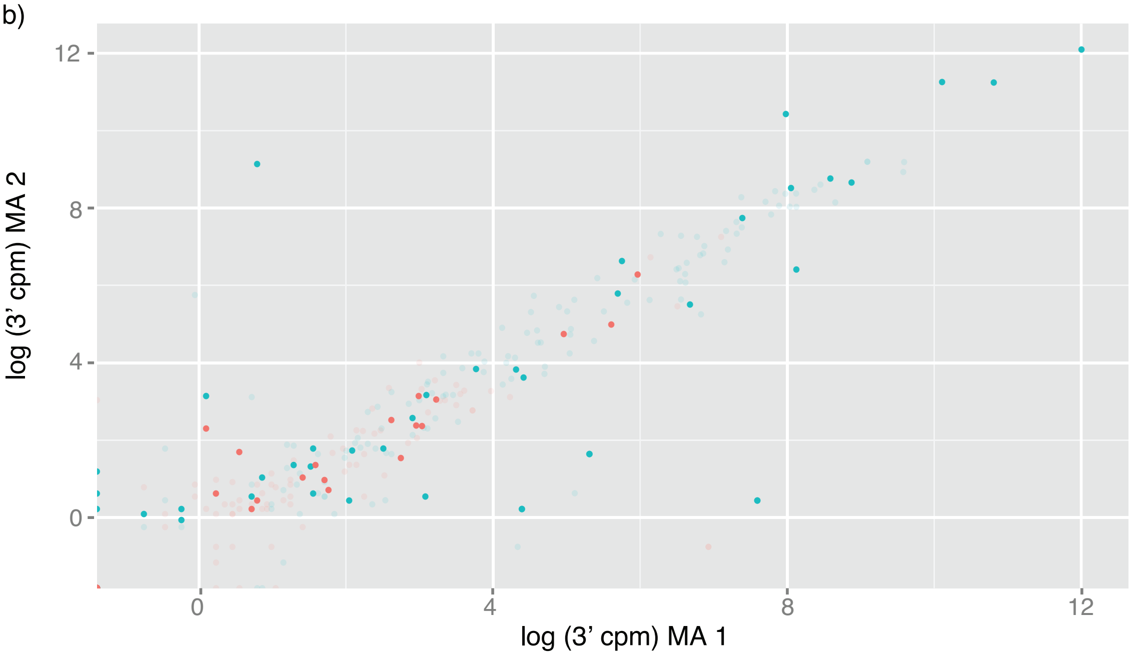

Supplementary Figure S4. Tag abundance in major ampullate (MA) glands from individual 1 versus individual 2. (a) All tags that matched a reference gene. (b) Tags that matched the 3'-most position of the sense strand (and the paired position of the antisense strand) of reference genes. Abundance is the natural log of the counts for that tag per million total tag counts (cpm). Tags that are significantly more or less abundant in MA glands than cephalothoraxes (FDR ≤ 0.05) are bolded. Tags that match the sense strand are turquoise and tags that match the antisense strand are salmon.

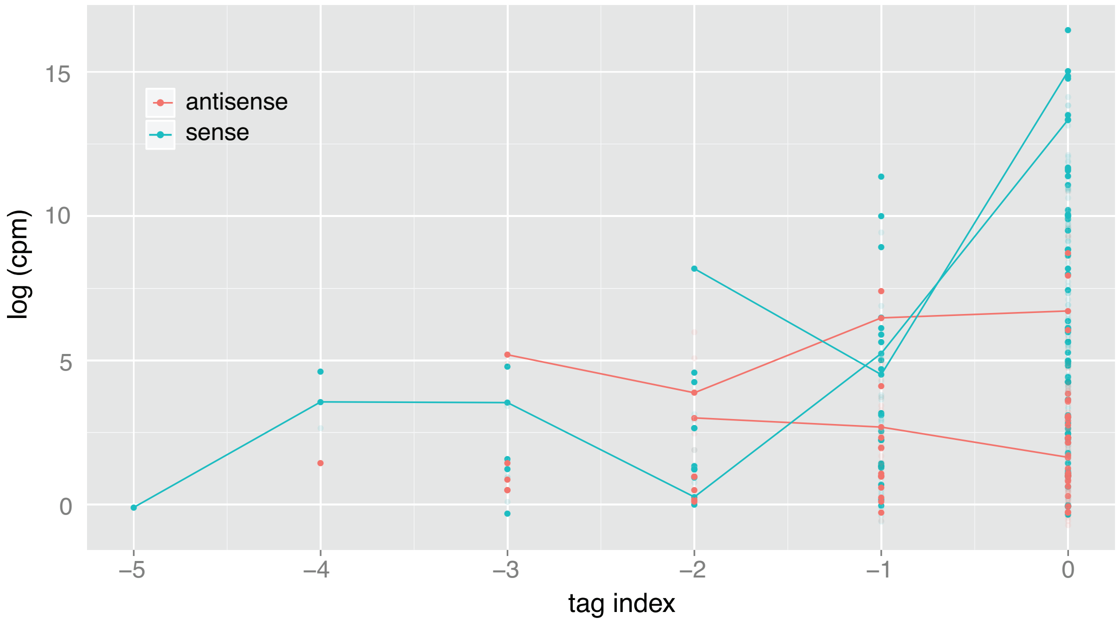

Supplementary Figure S5. Position of tag on a gene versus tag abundance for genes represented by more than one sense (turquoise) or antisense (salmon) tag. Tag abundance is the natural log of average counts of that tag per million total tag counts (cpm) among all four libraries. The tag index is the position of the tag relative to the 3' end of the sense strand with 0 being the 3'-most tag. Tags from *MaSp1* (DQ409057) and *MaSp2* (DQ409058) cDNAs are connected.

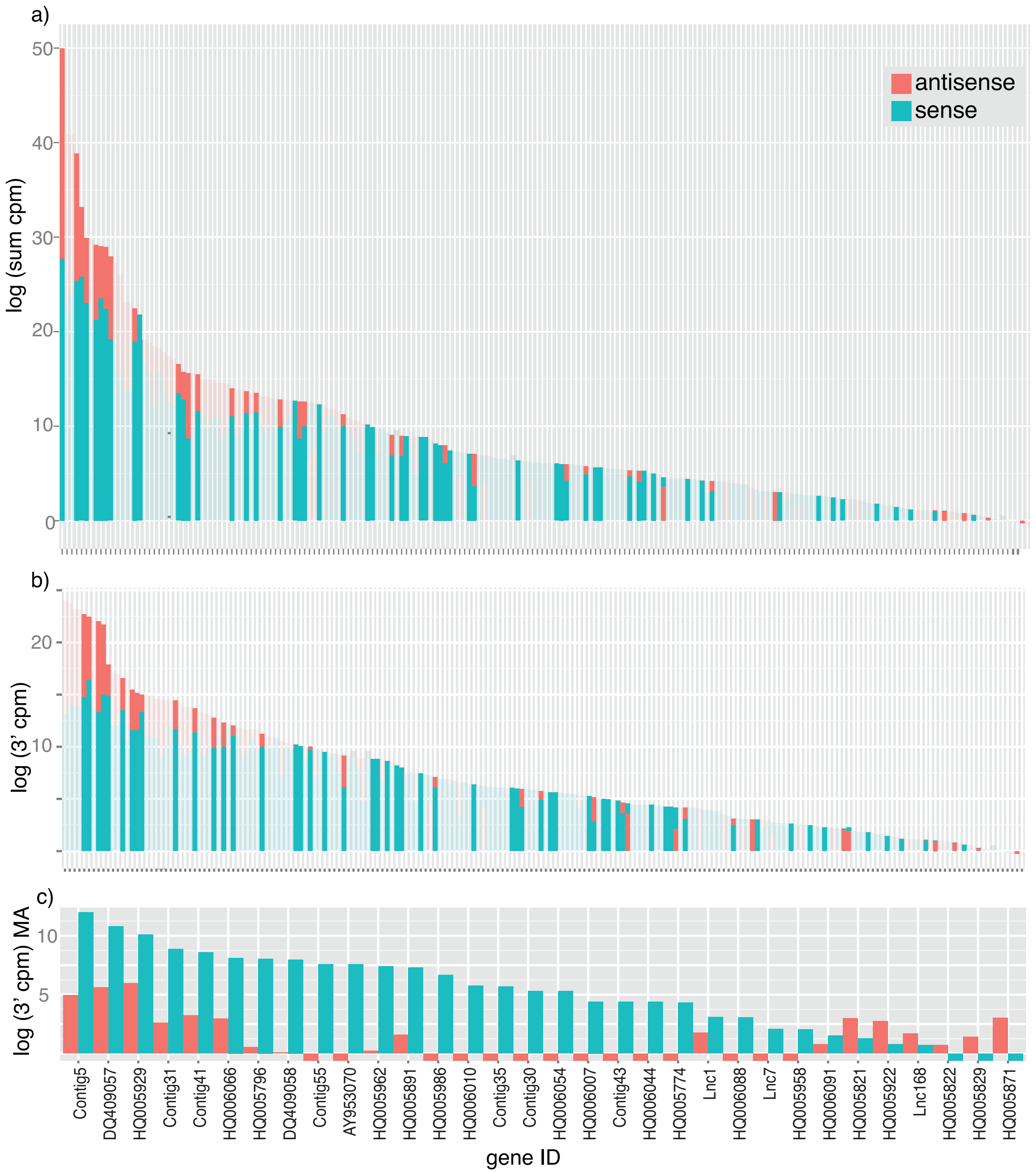

Supplementary Figure S6. Transcript abundance of black widow reference genes. (a) Total sense (turquoise) and antisense (salmon) abundance for all genes with an observed tag. Abundance is the sum of the average natural log (cpm), counts of that tag per million tags, of all tags that matched to that gene among all four libraries. (b) Abundance is estimated from only the 3'-most position of the sense strand or the matching position of the antinsense strand. (c) Abundance from MA glands based solely on the 3'-most tag for genes with a 3'-most tag that was more abundant in MA glands than cephalothoraxes (FDR ≤ 0.05).

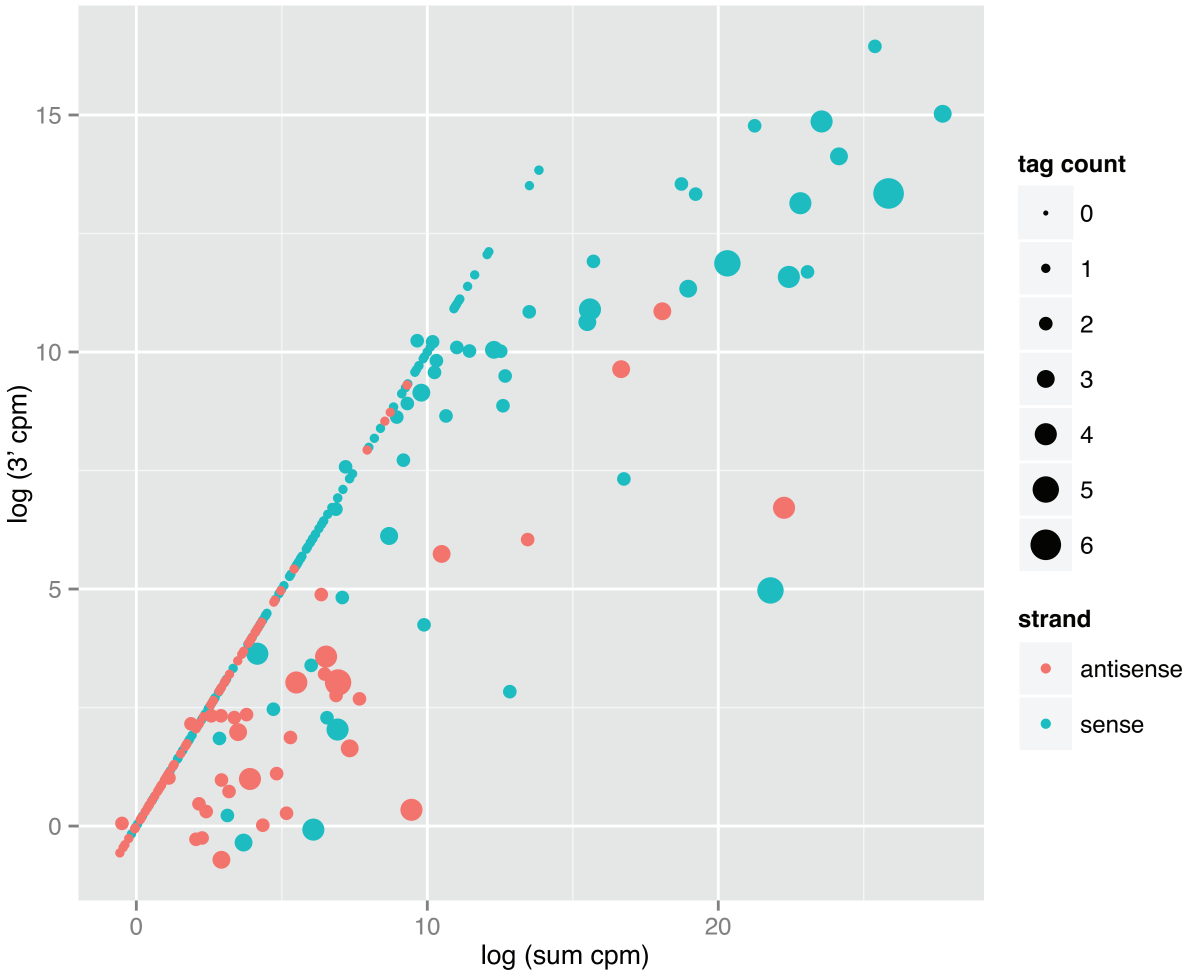

Supplementary Figure S7. Sense (turquoise) and antisense (salmon) transcript abundance estimated from the sum of tags that matched a reference gene versus abundance estimated from only the 3'-most position of the sense strand (or matched position of the antisense strand) of the reference genes. Tag abundance is the natural log of average counts of that tag per million total tag counts (cpm) among all four libraries. The two estimates are identical if only a single tag matched the sense or antisense strands of a reference gene (tag count = 1). Estimates of transcript abundance increase with increasing tag count (number of tags that match the sense or antisense strand of the reference gene), but are tightly correlated with the estimate based solely on the 3'-most tag.

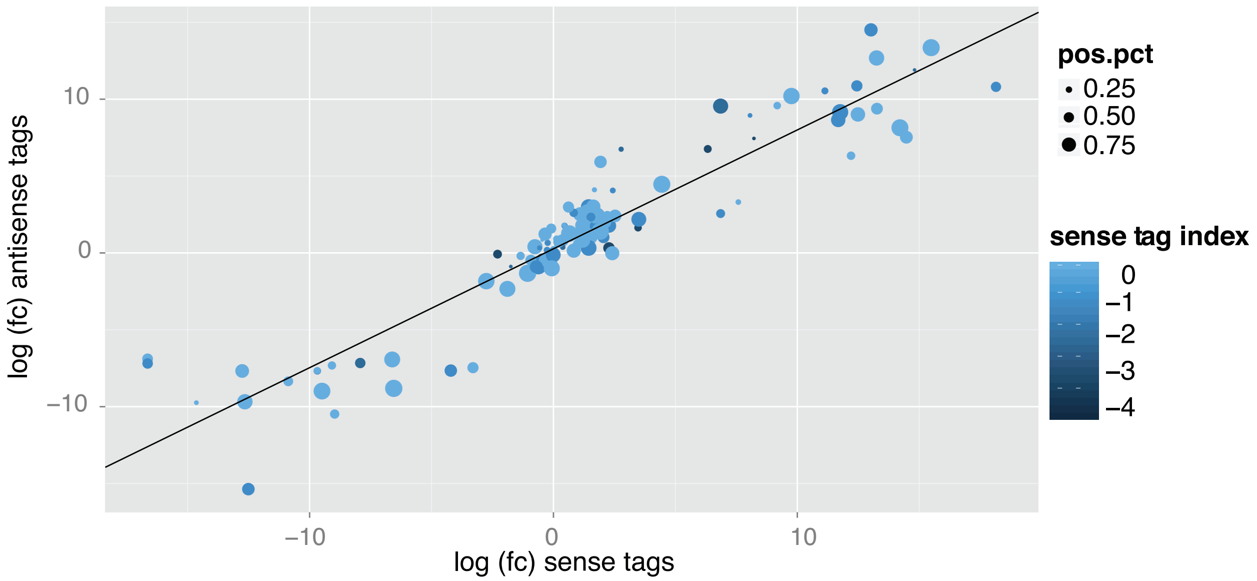

Supplementary Figure S8. Fold change, log (fc), between major ampullate glands and cephalothorax for sense versus antisense tags paired by position along gene. A positive log (fc) indicates higher abundance in major ampullate glands than cephalothoraxes. Position percent (pos.pct) is the position of the first base of the tag divided by the total length of the gene it matches to. Sense tag index indicates the position of the tag relative to other tags that match the same gene. The 3'-most tag is labeled with 0 with decreasing numbers towards the 5'-most tag. Best fit line is shown (slope = 0.77).

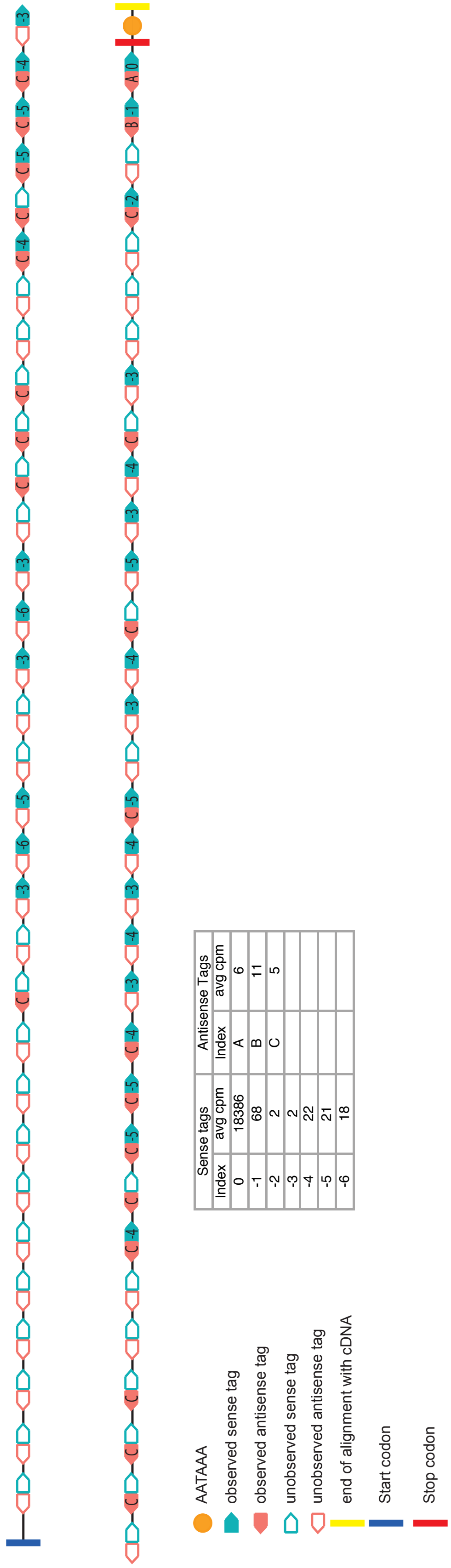

Sense tags		Antisense Tags	
Index	avg cpm	Index	avg cpm
0	18386	A	6
-1	68	B	11
-2	2	C	5
-3	2		
-4	22		
-5	21		
-6	18		

Supplementary Figure S9. **Diagram of unobserved (empty arrows) and observed (filled arrows) tags that match to the coding region of the full-length *MaSp2* gene sequence**. Antisense (salmon) and sense (turquoise) tags are paired by location. Observed unique tags are indexed by numbers (0 to -6, 3' to 5' respectively) for sense tags and lettered (A-C) for antisense tags. Abundance is the average counts of that tag per million total tags (cpm) in major ampullate glands of both individuals. The multiple occurrences of some tag sequences reflect the repetitive nature of *MaSp2*. Start codon (blue hatch), stop codon (red hatch), and the polyadenylation signal AATAAA (orange dot) are indicated. The cDNA aligns with the gene up to the yellow hatch. Location of tags is approximate and not to scale.

Supplementary Methods

R scripts

```
1) Backman_command_sequence
trimTags <- function(dgeReads) {
    # Purpose: This function trims the adapter tags from the dge (tag) reads

        # trim LR adapters
    seqs <- sread(dgeReads) # get sequence list
    qual <- quality(dgeReads) # get quality score list
    adapter <- "TCGTATGCCGTCTTCTGCTTGAAAAAAAAAA"
       # This is the adapter sequence to be trimmed from the end
    mismatchVector <- c(rep(2,length(DNAString(adapter))))
       # This is a vector of equal length to the adapter with "2" for each
value.
       #  This will allow the adapter to match the end of the sequence with
any offset
       #  and up to 2 mismatches.
    trimCoords <- trimLRPatterns(Rpattern=adapter, subject=seqs,
max.Rmismatch=mismatchVector, ranges=T)
       # Trim sequences looking for a right end pattern, requiring the first
4nt to match
       # Gets IRanges object with trimmed coordinates
    seqs <- DNAStringSet(seqs, start=start(trimCoords), end=end(trimCoords))
    qual <- BStringSet(quality(qual)) # strip quality score type
    qual <- BStringSet(qual, start=start(trimCoords), end=end(trimCoords))
       # Use IRanges coordinates to trim sequences and quality scores
    qual <- SFastqQuality(qual) # reapply quality score type
    trimmed <- ShortReadQ(sread=seqs, quality=qual, id=id(dgeReads))

    # trim size
    maxSize <- 17
    minSize <- 16
    trimmed <- trimmed[width(sread(trimmed)) <= maxSize] # remove all reads
longer than maxSize
    trimmed <- trimmed[minSize <= width(sread(trimmed))]
    return(trimmed)
}

# DGE (tag) profiling
samples <-
c("http://illumina.ucr.edu/illumina_runs/19/flowcell19_lane8.fastq",
"http://illumina.ucr.edu/illumina_runs/21/flowcell21_lane1.fastq",
"http://illumina.ucr.edu/illumina_runs/21/flowcell21_lane2.fastq",
"http://illumina.ucr.edu/illumina_runs/21/flowcell21_lane3.fastq")

# get into run folder
startDir <- getwd()
setwd(getConfigOption(configObject, "main", "workingPath"))



for(thisSample in samples) {
    # download data
    system(paste("wget",thisSample))
```

```
    thisSample <- gsub("^.*/(.*)$", "\\1", thisSample, perl="TRUE")

    dgeReads <- readFastq(thisSample)

    # filter reads
    passed <- grepl(":Y$", id(dgeReads), perl="TRUE")
    dgeReads <- dgeReads[passed]

    # trim reads
    dgeReads <- trimTags(dgeReads)

    # get first 16nt
    dgeReads <- sread(dgeReads)
    dgeReads <- DNAStringSet(dgeReads, end=16)

    # profile reads
    expressionProfile <- table(as.character(dgeReads))
    expressionProfile <- expressionProfile[expressionProfile > 1] # keep only
tags with expression > 1
    uniqueTags <- DNAStringSet(names(expressionProfile))
    names(uniqueTags) <- expressionProfile

    # export expression tag counts as fasta file
    OutputFilename <- paste("uniqueTagsFrom", thisSample, sep="")
    OutputFilename <- sub(".fastq$", ".fasta", OutputFilename)
    write.XStringSet(uniqueTags, OutputFilename)
}
```

2) Several parts of this script were adapted from “SequenceAnalysis” by Girke 2012 published at http://manuals.bioinformatics.ucr.edu/home/programming-in-r. This script inputs the sequences from our reference database and predicts every possible 20 base “tag”. It then inputs the cDNA library tags and compares them to the predicted tags. It then locates tags that are indentical in the two databases and outputs a table with the tag count data and the gene data from each database.

```
findTags<-function(fastaFile){

#Parses the fasta file into a usable table

  # reads file line-wise into vector
  my_fasta <- readLines(fastaFile)

  # identifies all fields that do not start with a '>' sign
  y <- regexpr("^[^>]", my_fasta, perl=T)
  y <- as.vector(y);  y[y==-1] <- 0
  index <- which(y==0)
  distance <- data.frame(start=index[1:(length(index)-1)],
end=index[2:length(index)])

  # gets data for last entry
  distance <- rbind(distance, c(distance[length(distance[,1]),2],
length(y)+1))
  distance <- data.frame(distance, dist=distance[,2]-distance[,1])
  seq_no <- 1:length(y[y==0])
  index <- rep(seq_no, as.vector(distance[,3]))
  my_fasta <- data.frame(index, y, my_fasta)
```

```
  my_fasta[my_fasta[,2]==0,1] <- 0
  seq <- tapply(as.vector(my_fasta[,3]), factor(my_fasta[,1]), paste,
collapse="", simplify=F)
  seq <- as.vector(seq[2:length(seq)])
  Desc <- as.vector(my_fasta[c(grep("^>", as.character(my_fasta[,3]), perl =
TRUE)),3])
  ID <- gsub("^>| .*", "", as.character(Desc), perl=T)
  Desc <- gsub("^.*? ", "", as.character(Desc), perl=T)
  my_fasta <- data.frame(ID, Desc, Length=nchar(seq), seq)

  #displays new table in order to check for accuracy
  View(my_fasta)

#creates the function to locate the pattern and place into appropriate
data.frame

  # expects sequences in fourth column of 'sequence' data frame
pattern_fct <- function(pattern, sequences) {

  # 'gregexpr' returns list with pattern positions
  pos <- gregexpr(pattern, as.character(sequences[,4]), perl=T)

  # retrieves positions where pattern matches in each sequence
  posv <- unlist(lapply(pos, paste, collapse=", ")); posv[posv==-1] <- 0

  # counts the number of hits per sequence
  hitsv <- unlist(lapply(pos, function(x) if(x[1]==-1) { 0 } else { length(x)
}))
  sequences <- data.frame(sequences[,1:3], Position=as.vector(posv),
Hits=hitsv, sequences[,4])
  }

#stores output from pattern_fct to use in next step
patterndf <- pattern_fct(pattern="GATC................", sequences=my_fasta)

#Shows output on screen to check for consistency
print(patterndf[patterndf[,5], 1:5])

#pulls up newest table
View(patterndf)

#creates open vectors for use in the next forloop
tags<-c()
genenames<-c()
ID<-c()
position<-c()

#takes columns from table that are factors and stores them as character
vectors
IDnames<-as.character(patterndf[,1])
geneInfo<-as.character(patterndf[,2])

#forloop which creates a numeric vector from the positions column row by row
and then works within each row to find tags

for(rowNumber in 1:nrow(patterndf)){

  positions<-patterndf[rowNumber,4]
```

```
  pV<-as.character(positions)
  cV<-strsplit(pV, ", ")
  uV<-unlist(cV)
  nV<-as.numeric(uV)


# forloop which takes each location within a tag and outputs the 16bp tag
without the "GATC"

  for(startPos in nV){
    if(startPos>0){
    bases<-substr(patterndf[rowNumber, 6], startPos+4, startPos+19)
    names<-geneInfo[rowNumber]
    codes<-IDnames[rowNumber]

    tags<-c(tags, bases)
    genenames<-c(genenames, names)
    ID<-c(ID, codes)
    position<-c(position, startPos)  } } }


#outputs your final table and brings it up
finalTable<-data.frame(ID=ID, genenames=genenames, tags=tags,
position=position)
View(finalTable)
return(finalTable)  }
```

4) This script is adapted from the EdgeR Analysis Protocol by Bioconductor found at http://www.bioconductor.org/packages/2.12/bioc/html/edgeR.html. It runs EdgeR by Bioconductor and performs the differential expression analysis on Tag libraries.

```
#This script performs analysis for differential expression on Tag libraries

# need a targets file with three columns: files, group, and description

#this creates a targets value
targets<-readTargets("targets.txt")
targets

#create a DGEList object for later use
d<-readDGE(targets, skip=5, comment.char = "!")
d$samples

#output a DGEList save file for originial library sizes
originalLibraryCounts<-d$sample
write.table(originalLibraryCounts, "Original_Library_Counts.txt", sep="\t")

#reports the number of unique tags, should record this value
dim(d)

#Filter low expression tags with an input of a cutoff value and the number of
libraries that must contain this number of hits
keep<-rowSums(cpm(d)>1)>=2
d<-d[keep,]

#reports the number of tags that fall within this accepted cutoff, should
record this value
```

```
dim(d)

#Since we have filtered out a large number of values we need to reset the
library sizes
d$samples$lib.size<-colSums(d$counts)
d$samples

#Calculate the TMM normalization factors for these new library sizes
d<-calcNormFactors(d)
d$samples

#output a DGEList save file for TMM normalization factors and library sizes
newLibraryCounts<-d$samples
newLibraryCounts<-data.frame(newLibraryCounts)
write.table(newLibraryCounts, "New_Library_Counts.txt", sep="\t")

#estimate the tagwise dispersions
d<-estimateTagwiseDisp(d, trend="none")

#conduct a tagwise test using the negative binomial test to determine
differential expression,

#enter the number of unique tags to retrieve all of the values in order
et<-exactTest(d)
topTags<-topTags(et, n= #number of unique tags )

#turn topTags into a data.frame to be able to use later
tagTable<-data.frame(topTags)

#retrieve number of hits per library as a data.frame
detags<-row.names(tagTable)
countTable<-cpm(d)[detags,]

#combine information from tagTable and countTable into a large data.fram to
output
fullTable<-cbind(tagTable, countsTable)


#save table as file
write.table(fullTable, "fullTable_8June2012.txt", sep="\t")

#perform summary analysis to see number of tags that are up, down, or
differentially expressed
dataSummary<-summary(de<-decideTestsDGE(et, p=0.05), adjust="BH")
dataSummary<-data.frame(summaryData)
write(dataSummary, "dataSummary.txt")
```
